# Early temperament as a predictor of language skills at 40 months

**DOI:** 10.1186/s12887-022-03116-5

**Published:** 2022-01-21

**Authors:** Yuuka Ishikawa-Omori, Tomoko Nishimura, Atsuko Nakagawa, Akemi Okumura, Taeko Harada, Chikako Nakayasu, Toshiki Iwabuchi, Yuko Amma, Haruka Suzuki, Mohammad Shafiur Rahman, Ryuji Nakahara, Nagahide Takahashi, Yoko Nomura, Kenji J. Tsuchiya

**Affiliations:** 1 Department of Child Development, United Graduate School of Child Development, Osaka University, Kanazawa University, Hamamatsu University School of Medicine, Chiba University, and University of Fukui, Handayama 1 Higashiku, Hamamatsu, 4313192 Japan; 2 Research Center for Child Mental Development, Hamamatsu University School of Medicine, Handayama 1 Higashiku, Hamamatsu, 4313192 Japan; 3 Graduate School of Humanities and Social Sciences, Nagoya City University, Mizuho-cho Yamanohata 1, Mizuho-ku, Nagoya, 4678501 Japan; 4 Department of Psychology, Queens College, City University of New York, 65-30 Kissena Blvd, Flushing, New York, NY 11367 USA; 5 Department of Psychiatry, Icahn School of Medicine at Mount Sinai, 1 Gustave L. Levy Place, New York, NY 10029 USA

**Keywords:** Language skills, Temperament, Cohort study, Early childhood behavior questionnaire (ECBQ)

## Abstract

**Background:**

Mastering language involves the development of expressive and receptive skills among children. While it has been speculated that early temperament plays a role in the acquisition of language, the actual mechanism has not yet been explored. We investigated whether temperament at 18 months predicted expressive or receptive language skills at 40 months.

**Methods:**

A representative sample of 901 children and their mothers who were enrolled and followed-up longitudinally in the Hamamatsu Birth Cohort for Mothers and Children study was included in the analysis. Child temperament was measured at 18 months using the Japanese version of the Early Childhood Behavior Questionnaire. Expressive and receptive language skills were measured at 40 months using the Mullen Scales of Early Learning.

**Results:**

The multiple regression analysis, adjusting for potential confounders, suggested that higher motor activation (fidgeting) at 18 months was associated with lower expressive and receptive language skills at 40 months. Higher perceptual sensitivity was associated with higher expressive and receptive language skills at 40 months.

**Conclusions:**

Specific temperament at 18 months of age predicted the development of the child’s expressive and receptive language skills at 40 months.

**Supplementary Information:**

The online version contains supplementary material available at 10.1186/s12887-022-03116-5.

## Background

Language plays a key role in understanding child neurodevelopment [1, 2]. Studies have suggested that language acquisition progresses significantly during the first 4 years of life [3], while language skills in early childhood are known to predict cognitive skills at school age [4] and academic performance in young adulthood [5]. Language skills in early childhood are influenced by individual characteristics [6, 7] of both the child and environment, including the family; for example, the child’s sex [8], birth weight [9], gestational age at birth [10], birth order [11], maternal age [12], maternal education [13], maternal history of mood or anxiety disorders [14, 15], and household income [8, 10]. Child temperament is also a candidate factor that can influence child language development [16–18].

In the literature, temperament has been understood as “the constellation of inborn traits that determine a child’s unique behavioral style” [19]. Along with this understanding, temperament has been measured in a few different ways. For instance, Goldsmith attempted to define it based on the emotionality of a child aged 16 to 36 months [20], although emotionality is not the only trait that determines an individual style of behavior; in particular, this is not the case for young children. In a hallmark study of temperament of young children, including those in the first year of life, Rothbart expanded the original concept and re-defined child temperament as individual differences in emotional, motor, and attentional reactivity and self-regulation [21]. Subsequent studies have suggested that temperamental characteristics appear as early as infancy and are driven by biological factors such as genes and brain structure [21, 22], out of which personality grows later in life [21]. Child temperament has also been studied as a predictor of physical and mental health problems and adjustment in later childhood and adolescence since it is observed early in development [21]. Among the many temperament scales developed [23], those developed by Rothbart and her colleagues, namely the Infant Behavior Questionnaire [24] for infants between 3 and 12 months of age and the Early Childhood Behavior Questionnaire (ECBQ; [25]) for children between 18 months and 3 years of age, are the most widely used parent-report measures assessing early childhood temperament [24]; these scales measure 21 fine-grained temperament dimensions [26]. Until recently, early child temperament has been reported to reflect early attentional skills, which is the basis for self-regulation [21, 27]. To this end, studies have shown that child language skills are associated with early attentional skills and self-regulation [28, 29], supporting the finding that temperament can be a determinant of language skills development [30]. In other words, exploring the association between child temperament and language skills development elucidates the mechanism of child language development.

Despite the growing recognition of child temperament’s role in language development in recent years, the link between them remains unclear (e.g., [16–18]). To our knowledge, four longitudinal studies have investigated whether child temperament was associated with language skill development using the temperament scale developed by Rothbart and colleagues [16, 17, 31, 32]. All four studies supported the association, but unclarity remained regarding which subscale of temperament was associated with language skills. Potential reasons behind this inconsistency include 1) the relatively modest sample sizes; for instance, the largest size was 148 in the study by Davison and colleagues [31], followed by 142 [17], 70 [32], and 47 [16]; 2) insufficient control of covariates including sex, birth weight, birth order, maternal age, maternal education, and household income; and 3) language skill was assessed only from the aspect of expressive skills (language productivity). Furthermore, several studies have reported associations between child temperament and expressive and receptive language skills during infancy and early childhood (e.g., [18, 33–36]). Unfortunately, these studies did not adopt scales developed by Rothbart [18, 33] and were cross-sectional [34–36]; thus, longitudinal studies are required to investigate whether the association is causal. To date, few studies have examined whether early temperament is related to language development using a longitudinal design (e.g., [16, 17, 31, 32]), and no specific subscales of temperament have been shown to be consistently associated with language development.

Due to the paucity and inconsistency of the findings thus far, we attempted to investigate whether child temperament at 18 months predicts expressive or receptive language skills at 40 months. To this end, we conducted an exploratory study with a relatively large sample size using a longitudinal design to evaluate the link between early temperament and expressive or receptive language skills, corresponding to language productivity and verbal comprehension, respectively. Furthermore, we controlled for covariates including sex, birth weight, birth order, maternal age, maternal education, and household income, which have previously been reported to be associated with language skills. As this study is exploratory, we treat all subscales equally without emphasizing specific subscales of temperament, considering the comparability of our findings to the existing literature.

## Methods

### Participants

The participants were mothers and their newborn infants, with mothers enrolled in the Hamamatsu Birth Cohort for Mothers and Children (HBC Study; [37, 38]) during pregnancy and infants being enrolled at birth. Both were monitored until the child was approximately 8 years old and continue to be monitored. Based on official national statistics, participants in this cohort are considered a representative sample of the general population of Japan; the enrolled mothers are representative of Japanese mothers with respect to age, socioeconomic status, and parity, and their children are representative of Japanese children with respect to birth weight and gestational age at birth [37, 38].

We contacted all the pregnant women (*N* = 1138) enrolled in the HBC Study who were expected to give birth, either at Hamamatsu University Hospital or Kato Maternity Clinic (both in Hamamatsu City), or who gave birth between December 24, 2007, and March 19, 2012. Nineteen mothers gave multiple births, 100 mothers entered the cohort twice, and one mother entered the cohort three times for a total of 1258 participating children. Moreover, 357 infants were excluded because of their absence at the 18 months examination when child temperament was measured or at the 40 months examination when language skills were measured, resulting in 901 out of 1258 infants (72%) being included in the current study.

The study was conducted in accordance with the guidelines of the World Medical Association Declaration of Helsinki and was approved by the Medical Ethical Committee of Hamamatsu University School of Medicine, Japan (No. 20–82, 21–114, 22–29, 24–67, 24–237, 25–143, 25–283, E14–062, 17–037, 17–037-3, 20–233). Written informed consent was obtained from all caregivers (mothers [except for three participating children], two grandmothers, and one father) for their own participation in the study and that of their infants.

### Materials and procedures

#### Child temperament

To measure child temperament, we administered the Japanese version of the ECBQ [25] at the age of 18 months. In a sample of Japanese children, Cronbach’s alpha coefficients of the 18 subscales ranged from 0.59 to 0.90 [39]. Both the Japanese version and original ECBQ included a parent-report questionnaire of 201 items and a three-factor structure of negative affectivity, effortful control, and surgency/extraversion [39]. Each of the three factors comprised several subscales, identical to the original ECBQ (Table 1). Our analyses focused on these 18 subscales and not on the three higher-order factors. This is because most of the existing literature used subscales (e.g., [16, 17, 31, 32]). Comparison with previous studies is easier by focusing on subscales than using higher-order factors. Each subscale described one specific aspect of behavior (e.g., “In a situation where s/he is meeting new people, how often did your child become quiet?” or “During everyday activities, how often did your child appear to listen to even very quiet sounds?”) and was rated on a 7-point scale ranging from “never” to “always,” depending on the frequency of its occurrence. To enhance the objectivity of parental assessments, caregivers were first asked to complete the ECBQ; hereafter, a face-to-face interview was conducted with them by an examiner of our research team.

**Table 1:** Definition of subscales: Early Childhood Behavior Questionnaire (ECBQ)

Factor/Subscales	Definition
Negative affectivity	
Discomfort	Amount of negative affect related to sensory qualities of stimulation.
Fear	Negative affect related to anticipated pain, distress, sudden events and/or potentially threatening situations.
Frustration	Negative affect related to interruption of ongoing tasks or goal blocking.
Motor activation	Repetitive small-motor movements; fidgeting.
Perceptual sensitivity	Detection of slight, low-intensity stimuli from the external environment.
Sadness	Tearfulness or lowered mood related to suffering, disappointment, or loss.
Shyness	Slow or inhibited approach and/or discomfort in social situations involving novelty or uncertainty.
Soothability	Rate of recovery from peak distress, excitement, or general arousal.
Effortful control	
Attentional focusing	Sustained duration of orienting on an object of attention; resisting distraction.
Attentional shifting	The ability to transfer attentional focus from one activity/task to another.
Cuddliness	Child’s expression of enjoyment in and molding of the body to being held by a caregiver.
Inhibitory control	The capacity to stop, moderate, or refrain from a behavior under instruction.
Low-intensity pleasure	Pleasure or enjoyment related to situations involving low intensity, rate, complexity, novelty, and incongruity.
Surgency/extraversion	
Activity level	Level (rate and intensity) of gross motor activation, including rate and extent of locomotion.
High-intensity pleasure	Pleasure or enjoyment related to situations involving high intensity, rate, complexity, novelty, and incongruity.
Impulsivity	Speed of response initiation.
Positive anticipation	Excitement about expected pleasurable activities.
Sociability	Seeking and taking pleasure in interactions with others.

Note. Adapted from “Measurement of fine-grained aspects of toddler temperament: The Early Childhood Behavior Questionnaire,” by Putnam SP, Gartstein MA, and Rothbart MK, Infant Behavior Development 2006; 29(3), p. 399 (10.1016/j.infbeh.2006.01.004). Copyright 2006 by Elsevier Inc

#### Expressive and receptive language skills

The assessment of language development in early childhood requires evaluation of both expressive and receptive language skills [3]. We used two subscales of the Mullen Scales of Early Learning (MSEL; [40]). In the MSEL, expressive language skills are defined as those that support the ability to use language productively, such as speaking, and receptive language skills are defined as those that promote processing of linguistic input, such as verbal comprehension [40, 41]. We previously developed z-scores for each MSEL subscale in Japanese children using our HBC Study sample, as the US version of the normative data did not correspond well with the Japanese sample [42]. In the current study, we used the standardized version for Japanese children, in which the internal consistency of expressive and receptive language scores at 40 months was alpha = 0.84, comparable to the original US version. The z-scores had a mean of 0 and a standard deviation of 1 and were derived from the standardized scores prepared in our previous study [42].

#### Covariates

Sex [8], birth weight [9], gestational age at birth [10], birth order [11], maternal age at childbirth [12], maternal education [13], household income at birth [8, 10], and maternal history of mood or anxiety disorders [14, 15], previously reported to be associated with language skills in general, were treated as covariates. Information on the demographic and socioeconomic characteristics of the mothers (maternal age, years of maternal education, annual household income, and maternal history of mood or anxiety disorder) was collected through face-to-face interviews during the second trimester of the index pregnancy and in the first 1 to 2 months after childbirth. Information on the infants’ sex, gestational age, birth weight, and date of birth was obtained directly from medical records.

#### Analyses

Multiple regression analyses were performed to examine the associations between each of the 18 subscales of the ECBQ and the expressive and receptive language scales at 40 months. First, univariate regression analyses were performed to test the association of each subscale with expressive and receptive language scores (Model 1). Hereafter, all 18 subscale scores were analyzed simultaneously in a single model (Model 2). Finally, covariates were added to the model (Model 3). We did not remove statistically non-significant covariates from Model 3 because it is recommended that, in studies exploring risk factors, any established risk factors for the outcome should be retained in the model regardless of the statistical significance [43].

Stata version 13.1 was utilized for all analyses. We used *p*-values to analyze the data, as these are meaningful in exploratory analysis [44]. Considering the adjustments for multiple testing, we defined *p*-values < .0013 (= .05/36) to be statistically significant to allow for measurements of 18 temperamental subscales and both language skills (i.e., expressive and receptive language).

## Results

### Participant characteristics

Table 2 shows the demographic characteristics of the children and their parents included in the analysis. Comparisons of the 901 participants in the final analytic sample and the 357 children excluded from the analyses revealed no significant differences in sex, birth weight, gestational age at birth, distribution of birth order, nor twin births (see Supplementary Table 1). However, when comparing the mothers in the two groups, the following significant differences were found: the mean age of the mothers at the child’s birth was significantly lower among excluded children (M = 30.4, SD = 5.0) than participating children (M = 31.9, SD = 5.0; z = − 4.33, *p* < .001) and annual household income was significantly lower among excluded children (M = 5.78, SD = 2.84 million JPY) than participating children (M = 6.13, SD = 2.82 million JPY; z = − 2.36, *p* = .02).

**Table 2:** Demographic characteristics of the children and parents included in the analysis (*N* = 901)

	n (%) or M (SD)	Range
Child sex (Boys %)	450 (50%)	
Birthweight (g)	2931 (441)	1064–4286
Gestational age at birth (weeks)	38.9 (1.6)	29.6–42.1
Birth order		
First	447 (50%)	
Second	337 (37%)	
Third or Later	117 (13%)	
Twin births	29 (3%)	
Age of mother at the time of the child’s birth (years)	31.9 (5.0)	17.7–44.9
Mother’s education (years)	13.9 (1.9)	6.0–23.0
History of maternal psychiatric diagnosis (Yes %)	90 (10%)	
Annual household income (million JPY)	6.13 (2.82)	1.0–27.0
The ECBQ scores at 18 months of age		
Discomfort	2.0 (0.7)	1.0–5.2
Fear	2.3 (0.7)	1.0–5.1
Frustration	3.3 (0.9)	1.0–7.0
Motor activation	2.2 (0.8)	1.0–5.5
Perceptual sensitivity	3.3 (1.0)	1.0–6.4
Sadness	2.5 (1.0)	1.0–6.0
Shyness	3.5 (1.1)	1.0–6.8
Soothability	5.7 (0.9)	1.5–7.0
Attentional focusing	3.7 (1.1)	1.0–6.8
Attentional shifting	4.6 (0.8)	1.8–6.9
Cuddliness	4.1 (1.1)	1.5–7.0
Inhibitory control	3.1 (0.9)	1.0–6.1
Low-intensity pleasure	4.7 (0.8)	1.8–6.7
Activity level	4.9 (0.8)	2.1–7.0
High-intensity pleasure	4.2 (1.6)	1.0–7.0
Impulsivity	4.3 (1.3)	1.0–7.0
Positive anticipation	3.9 (0.9)	1.0–5.0
Sociability	4.7 (1.9)	1.0–7.0
Expressive language scores (z-score)	−0.01 (1.02)	−3.00–2.88
Receptive language scores (z-score)	−0.02 (0.98)	−3.00–3.00

Note. *ECBQ* Early Child Behavior Questionnaire

All 18 ECBQ subscales were graded from 1 to 7, with some variations in the mean values. We found correlations among the 18 subscales (see Supplementary Table 2). The mean z-scores of expressive and receptive language skills at 40 months were all close to 0, but not exactly 0. This was expected because, in our analysis, we excluded 357 children from the original sample of the HBC Study, from which the Japanese version of the normative data were derived.

### ECBQ subscales and expressive language skills at 40 months

Table 3 shows the regression coefficients and 95% confidence intervals for each of the subscales in Models 1, 2, and 3 (all the coefficients estimated in Models 1 to 3 are shown in Supplementary Table 3). In Model 1 (univariate), six subscales were significantly associated with expressive language scores. However, in Model 2 (controlling for all 18 subscale scores), only two subscales were significantly associated with expressive language scores, while the remaining subscales were nonsignificant. After controlling for all covariates (Model 3), the motor activation subscale was negatively associated with expressive language, and the perceptual sensitivity subscale was positively associated with expressive language.

**Table 3:** Associations of subscales of the Early Child Behavior Questionnaire and expressive language scores at 40 months: regression coefficients in z-score (change in SD), 95% confidence intervals and *p*-values

	Model 1		Model 2		Model 3	
	Coefficient [95%CI]	*p*	Coefficient [95%CI]	*p*	Coefficient [95%CI]	*p*
Motor activation	**−0.192** [−0.274, − 0.109]	< .001	**−0.234** [− 0.331, − 0.137]	< .001	**− 0.211** [− 0.305, − 0.117]	< .001
Perceptual sensitivity	**0.144** [0.079, 0.209]	< .001	**0.157** [0.079, 0.236]	< .001	**0.137** [0.061, 0.213]	< .001
Inhibitory control	**0.190** [0.115, 0.265]	< .001	0.110 [0.024, 0.195]	.01	0.091 [0.009, 0.173]	.029
Soothability	**0.119** [0.048, 0.189]	.001	0.028 [−0.055, 0.110]	.51	0.024 [− 0.054, 0.103]	.55
Attentional shifting	**0.232** [0.146, 0.317]	< .001	0.095 [−0.005, 0.194]	.06	0.087 [−0.008, 0.182]	.07
Low-intensity pleasure	**0.206** [0.116, 0.296]	< .001	0.105 [0.008, 0.202]	.034	0.066 [−0.026, 0.159]	.16

Note. Model 1 = Univariate; Model 2 = Adjusted for other subscales of the Early Child Behavior Questionnaire Subscales (ECBQ); Model 3 = Model 2 with further adjustment for child sex, birth weight, gestational age at birth, birth order, age of the mother, years of maternal education, annual household income, maternal history of mood/anxiety disorders. *CI* Confidence intervals. Bold types represent *p* < .0013. The regression coefficients shown in Table 3 indicate the amount of predicted change in the z-score of expressive language skills per one unit of change in each of the ECBQ subscales. The marginal magnitude of difference was calculated as follows: a child scoring 7 (maximum) in motor activation, for example, was predicted to score − 0.211 × 6 (i.e., 7 minus 1 point in the subscale) = − 1.266 points, corresponding to − 1.266 SD higher lower in the expressive language skill score at 40 months compared with a child scoring 1 (minimum)

### ECBQ subscales and receptive language skills at 40 months

Table 4 shows the regression coefficients and 95% confidence intervals for each of the subscales in Models 1, 2, and 3 (all the coefficients estimated in Models 1 to 3 are shown in Supplementary Table 4). In Model 1, four subscales were significantly associated with receptive language scores. In Model 2, two subscales (motor activation and perceptual sensitivity) were significantly associated with receptive language scores, while all other associations were nonsignificant. Even after controlling for all covariates (Model 3), two subscales remained significantly associated with receptive language scores: the motor activation subscale was negatively associated with receptive language scores, and the perceptual sensitivity subscale was positively associated with receptive language scores.

**Table 4:** Associations of subscales of the Early Child Behavior Questionnaire and receptive language scores at 40 months: regression coefficients in z-score (change in SD), 95% confidence intervals and *p*-values

	Model 1		Model 2		Model 3	
	Coefficient [95%CI]	*p*	Coefficient [95%CI]	*p*	Coefficient [95%CI]	*p*
Motor activation	**−0.174** [− 0.254, − 0.095]	< .001	**− 0.245** [− 0.339, − 0.150]	< .001	**−0.225** [− 0.317, − 0.133]	< .001
Perceptual sensitivity	**0.132** [0.069, 0.195]	< .001	**0.169** [0.092, 0.245]	< .001	**0.150** [0.076, 0.225]	< .001
Attentional shifting	**0.178** [0.095, 0.262]	< .001	0.071 [−0.026, 0.168]	.15	0.063 [−0.030, 0.156]	.18
Inhibitory control	**0.145** [0.072, 0.218]	< .001	0.082 [−0.001, 0.165]	.05	0.066 [−0.015, 0.146]	.11

Note. Model 1 = Univariate; Model 2 = Adjusted for other subscales of the Early Child Behavior Questionnaire Subscales (ECBQ); Model 3 = Model 2 with further adjustment for child sex, birth weight, gestational age at birth, birth order, age of the mother, years of maternal education, annual household income, maternal history of mood/anxiety disorders. *CI* Confidence intervals. Bold types represent *p* < .0013. The regression coefficients shown in Table 4 indicate the amount of predicted change in the z-score of receptive language skills per one unit of change in each of the ECBQ subscales. Again, this implies that a child scoring 7 (maximum) in motor activation, for example, was predicted to score − 0.225 × 6 (7 minus 1 point in the subscale) = − 1.350 points, corresponding to − 1.350 SD higher lower in the receptive language skills score at 40 months compared with a child scoring 1 (minimum)

## Discussion

This study investigated whether child temperament at 18 months of age, measured with the ECBQ, predicted expressive and receptive language skills at 40 months. The motor activation and perceptual sensitivity temperament subscales at 18 months predicted both expressive and receptive language skills at 40 months.

### Motor activation subscale and language skills

We found that higher scores on the motor activation subscale predicted lower scores for both expressive and receptive language skills. This could be explained by the subscale being defined as fidgeting (Table 1), as children who fidget repetitively at 18 months are assumed to generally have limited language skills during early childhood. This is consistent with the observation provided by Garello et al. [45], who demonstrated that high scores on the motor activation subscale at 24–30 months were associated with lower expressive language skills when measured simultaneously. In addition, Berger et al. [46] found that poor motor adjustment during early childhood, a representation of poor ability to sit still, required early attentional skills for performing tasks at hand. This makes it difficult for children to learn language skills [47]. We have expanded the work done by Garello et al. [45] by providing evidence that fidgeting behavior at 18 months, perhaps due to the cost of attentional resources, predicts poorer language skills at 40 months.

### Perceptual sensitivity subscale and language skills

Higher scores on the perceptual sensitivity subscale predicted higher scores for both expressive and receptive language skills at 40 months. Perceptual sensitivity is a construct that reflects the ability to detect slight, low-intensity stimuli in the external environment (Table 1). Specifically, such stimuli in the external environment cover several modality areas including auditory, visual, and tactile stimuli, although most of the questions categorized in this ECBQ subscale are related to auditory stimuli [25]. Notably, it is suggested that music, a broad set of patterned auditory stimuli, enhances speech perception in infancy [48], which predicts expressive and receptive language skills in later years [49]. It is likely that children exhibiting better performance in auditory perception achieve advanced language skills in later years. Furthermore, studies have consistently suggested that perceptual sensitivity is a reflection of early attentional skills [25, 50], which drive children to selectively attend to subtle stimuli and respond to them with their voices [51]. To this end, we presume that children with higher scores on the perceptual sensitivity subscale can better attend to and handle external stimuli, particularly auditory stimuli, which is reflected in their higher scores in language skills during early childhood.

### Relevance of early attention to the findings

Higher scores in motor activation and lower scores in perceptual sensitivity both accompanied problems in early attentional skills. The original studies indicated that attentional skills might emerge as early as the first year of life [21] and serve as a foundation of behavioral control over reactivity [51]. As such, higher scores in motor activation subscale involve difficulties in sitting still [46], and lower scores in perceptual sensitivity subscale involve poor response to external stimuli, including human voices [51]. These examples highlight the significant role of early attentional skills in language learning [30, 52].

### Subscales that have no association with language skills

Unlike the relevance of early attentional skills to the two subscales (motor activation and perceptual sensitivity) that predicted language skills at 40 months, other attention-related subscales (attentional focusing and attentional shifting subscales) did not similarly predict language skills. To illustrate, the two subscales were marginally or significantly associated with both expressive and receptive language skills at 40 months only in Model 1, although the associations became insignificant after adjusting for the other subscales (Supplementary Tables 3 and 4). Our findings are inconsistent with several studies reporting the relevance of two attentional skills (attentional focusing and shifting) to language development [53], although the methodologies employed were different. More importantly, studies by the developers of ECBQ have shown that the attentional focusing and shifting subscales are categorized as “executive attention” and that these two skills emerge as an early stage of development in self-regulation prominent in later years (approximately 4 years) [25, 27, 29]. Executive attention was defined as “mechanisms for monitoring and resolving conflict among thoughts, feelings, and responses” [54] and was suggested to form the basis of executive function [55]. Thus, the attentional focusing and shifting subscales may not be interpreted in the same way as the motor activation and perceptual sensitivity subscales representing early attention emerging during the first year of life. In other words, the association of early temperament and later language skills is age-specific; while the attentional focusing and shifting subscales at 18 months are not predictors for language skills at 40 months, the motor activation and perceptual sensitivity subscales at 18 months specifically predict level of language skills at 40 months.

### Other factors that may explain our findings

External factors should also be considered when interpreting our results. That is, language skills during early childhood have been reported to be influenced by child factors such as sex [8], birth weight [9], gestational age at birth [10], and birth order [11]. Maternal factors, such as age [12], years of education [13], and history of mood or anxiety disorders [14, 15] have also been found to influence language skills during early childhood. Even after controlling for these factors, the associations remained significant (see Supplementary Tables 3 and 4). This suggests that the associations between specific child temperament and language skills are generally independent of known external factors, although we did not control for all external factors (e.g., attachment [56] and parenting [57]).

### Clinical implications and future directions

Lower scores on the motor activation subscale and higher scores on the perceptual sensitivity subscale predict advantageous courses in language development in expressive and receptive skills. These findings are beneficial for child health professionals to predict sufficient language development during early childhood and to identify children at potential risk of insufficient language development. This is because early child temperament can be easily measured without any expertise and with a range of available tools, including the full and short versions of the ECBQ [58].

Future studies are expected to elucidate potential biological mechanisms underlying the association between child temperament and language skills. Genetic factors may be one account for the associations we observed. Although genes that account for the association between temperament and language skills have not yet been reported, expressive and receptive language skills are influenced by genetic factors [59]; child temperament is also genetically determined [22].

### Limitations and strengths

The present study has several limitations that should be considered when interpreting the results. First, approximately 28% of participants were excluded from the analysis, although comparisons between the analyzed and excluded participants did not reveal significant differences except for mothers’ age and household income. Second, we conducted conservative hypothesis tests. While one may set the significance level more loosely using, e.g., the false discovery rate, we did not do this because of our explorative study design which did not propose any hypothesis in advance. Third, although we considered a range of potential confounders or covariates, other variables that could have been controlled for, for example, attachment [56], parenting [57], family history of language delay [13], child-directed speech [60], and home literacy environment [61] were not measured, as their roles were not pivotal (e.g., [32, 62]). Fourth, the ECBQ is a parent-reported questionnaire, which may have led to information bias. For example, parents whose children showed language delays might have given lower scores on the ECBQ. To minimize this bias, we confirmed the inputs from the caregivers by conducting interviews with them. Finally, child temperament measures using the ECBQ were assessed at one time point.

Despite these limitations, the strengths of this study include its longitudinal design with a larger sample size compared to existing studies. Moreover, it focused on each subscale of the ECBQ while controlling for other subscales and covariates that have been found to relate to temperament and language.

## Conclusions

The present study found that specific temperamental subscales—motor activation and perceptual sensitivity—at 18 months of age predicted language skills at 40 months of age. These results remained significant even when controlling for other potential confounding factors, suggesting the relevance of specific temperament in language development and that these temperament subscales are useful for predicting language development in children. Taken together, our findings show that temperament, especially in areas of motor activation and perceptual sensitivity, can aid clinicians in evaluating the potential risk of insufficient language development later in children and provide opportunities for early interventions.

## Supplementary Information

**Additional file 1: Supplementary Table 1.** Demographic characteristics of the children and parents excluded in the analysis. **Supplementary Table 2.** The Pearson correlation matrix of the 18 subscales of the Early Child Behavior Questionnaire. **Supplementary Table 3.** Associations of 18 subscales of the Early Child Behavior Questionnaire and expressive language scores at 40 months: regression coefficients in z-score, 95% confidence intervals and *p*-values. **Supplementary Table 4.** Associations of 18 subscales of the Early Child Behavior Questionnaire and receptive language scores 40 months: regression coefficients in z-score, 95% confidence intervals and *p*-values.

## Data Availability

The analysis code compiled using Stata software is available from the corresponding author. Data sharing with readers is not available because of the requirements of the local Ethical Review Board.

## Abbreviations

ECBQ: Early Childhood Behavior Questionnaire
HBC Study: Hamamatsu Birth Cohort for Mothers and Children
MSEL: Mullen Scales of Early Learning
