# Supplementary material for: Early temperament as a predictor of language skills at 40 months
Source: BMC Pediatr. 2022 Jan 21;22:56. doi: 10.1186/s12887-022-03116-5 (PMC8780364; doi:10.1186/s12887-022-03116-5)
Supplement: Supplementary file 1 — Additional file 1: Supplementary Table 1. Demographic characteristics of the children and parents excluded in the analysis. Supplementary Table 2. The Pearson correlation matrix of the 18 subscales of the Early Child Behavior Questionnaire. Supplementary Table 3. Associations of 18 subscales of the Early Child Behavior Questionnaire and expressive language scores at 40 months: regression coefficients in z-score, 95% confidence intervals and p-values. Supplementary Table 4. Associations of 18 subscales of the Early Child Behavior Questionnaire and receptive language scores 40 months: regression coefficients in z-score, 95% confidence intervals and p-values. [file 12887_2022_3116_MOESM1_ESM.docx]

**Supplementary Table 1** Demographic characteristics of the children and parents excluded in the analysis

|  | Analyzed participants (n = 901) | | Excluded participants (n = 357) | | Statistics ^a^ |
| --- | --- | --- | --- | --- | --- |
|  | n (%) or M (SD) | Range | n (%) or M (SD) | Range |  |
| Child sex (Boys %) | 450 (50%) |  | 198 (55%) |  | χ２(1) = 3.12, p = .08 |
| Birthweight (g) | 2931 (441) | 1064–4286 | 2,963 (433) | 946–4,166 | z = 1.16, p = .24 |
| Gestational age at birth (weeks) | 38.9 (1.6) | 29.6–42.1 | 38.9 (1.6) | 28.1–42.1 | z = 0.26, p=. 80 |
| Apgar scores (at 5 minutes) | ^b^ 9.2 (0.7) | 0–10 | ^c^ 9.1 (1.1) | 0–10 | z = 0.37, p = .71 |
| Birth order |  |  |  |  | χ２(2) = 0.123, p = .94 |
| First | 447 (50%) |  | 179 (50%) |  |  |
| Second | 337 (37%) |  | 130 (36%) |  |  |
| Third or later | 117 (13%) |  | 48 (13%) |  |  |
| Twin births | 29 (3%) |  | 9 (3%) |  | χ２(1) = 0.425, p = .52 |
| Age of mother at the time of the children’s birth (years) | 31.9 (5.0) | 17.7–44.9 | 30.4 (5.0) | 18.0–40.6 | z = −4.33, p＜.001 |
| Mother's education (years) | 13.9 (1.9) | 6.0–23.0 | 13.6 (2.1) | 7.0–23.0 | z = −1.65, p =.10 |
| History of maternal psychiatric diagnosis (Yes %) | 90 (10%) |  | 33 (9%) |  | χ２(1) = 0.161, p = .70 |
| Household income (million JPY) | 6.13 (2.82) | 1.0–27.0 | 5.78 (2.84) | 0.79–23.0 | z = −2.36, p =.02 |
| Mother's marital status ^d^ (married %) | 892 (99%) |  | 349 (98%) |  | χ２(2) = 2.96, p = .08 |
| Expressive language scores (z-score) | −0.01 (1.02) | −3.00–2.88 | ^e^ −0.44 (1.12) | −3.00–2.27 | t (936) = −2.51, p = .01 |
| Receptive language scores (z-score) | −0.02 (0.98) | −3.00–3.00 | ^f^ −0.31 (0.96) | −3.00–2.08 | t (942) = −1.89, p = .06 |

Note. ^a^ Comparisons between the 901 analyzed participants and 357 excluded participants, ^b^ n=889, ^c^ n=347, ^d^ Mother's marital status from pregnancy to 6 months after childbirth,

^e^ n=37, ^f^ n=43

**Supplementary Table 2** The Pearson correlation matrix of the 18 subscales of the Early Child Behavior Questionnaire (N=901)

|  | 1 | 2 | 3 | 4 | 5 | 6 | 7 | 8 | 9 |
| --- | --- | --- | --- | --- | --- | --- | --- | --- | --- |
| 1 Discomfort | – |  |  |  |  |  |  |  |  |
| 2 Fear | 0.48** | – |  |  |  |  |  |  |  |
| 3 Frustration | 0.24** | 0.23** | – |  |  |  |  |  |  |
| 4 Motor activation | 0.33** | 0.24** | 0.28** | – |  |  |  |  |  |
| 5 Perceptual sensitivity | 0.44** | 0.22** | 0.12** | 0.34** | – |  |  |  |  |
| 6 Sadness | 0.24** | 0.25** | 0.43** | 0.32** | 0.14** | – |  |  |  |
| 7 Shyness | 0.22** | 0.44** | 0.08* | 0.11** | 0.12** | 0.20** | – |  |  |
| 8 Soothability | −0.21** | −0.28** | −0.35** | −0.30** | −0.07* | −0.40** | −0.26** | – |  |
| 9 Attentional focusing | −0.00 | 0.03 | −0.10** | −0.08* | 0.10** | −0.03 | 0.02 | −0.02 | – |
| 10 Attentional shifting | 0.05 | 0.02 | −0.06 | 0.01 | 0.31** | −0.11** | −0.06 | 0.25** | 0.17** |
| 11 Cuddliness | −0.15** | −0.02 | −0.18** | −0.19** | −0.11** | −0.09** | 0.11** | 0.04 | 0.06 |
| 12 Inhibitory control | −0.01 | −0.02 | −0.30** | −0.22** | 0.11** | −0.14** | −0.03 | 0.21** | 0.16** |
| 13 Low-intensity pleasure | 0.00 | 0.03 | −0.09** | −0.14** | 0.05 | −0.05 | 0.08* | 0.13** | 0.18** |
| 14 Activity level | 0.10** | ­−0.00 | 0.26** | 0.26** | 0.07* | 0.10** | −0.16** | ­−0.02 | −0.22** |
| 15 High-intensity pleasure | 0.18** | −0.01 | 0.13** | 0.19** | 0.20** | 0.15** | −0.06 | 0.00 | −0.10** |
| 16 Impulsivity | 0.03 | −0.14** | 0.11** | 0.14** | 0.04 | 0.02 | −0.42** | 0.11** | −0.16** |
| 17 Positive anticipation | 0.07* | 0.06 | 0.19** | 0.18** | 0.13** | 0.18** | −0.01 | −0.08* | −0.03 |
| 18 Sociability | 0.10** | 0.01 | 0.06 | 0.04 | 0.13** | 0.03 | −0.10** | 0.09** | 0.01 |

|  | 10 | 11 | 12 | 13 | 14 | 15 | 16 | 17 | 18 |
| --- | --- | --- | --- | --- | --- | --- | --- | --- | --- |
| 1 Discomfort |  |  |  |  |  |  |  |  |  |
| 2 Fear |  |  |  |  |  |  |  |  |  |
| 3 Frustration |  |  |  |  |  |  |  |  |  |
| 4 Motor activation |  |  |  |  |  |  |  |  |  |
| 5 Perceptual sensitivity |  |  |  |  |  |  |  |  |  |
| 6 Sadness |  |  |  |  |  |  |  |  |  |
| 7 Shyness |  |  |  |  |  |  |  |  |  |
| 8 Soothability |  |  |  |  |  |  |  |  |  |
| 9 Attentional focusing |  |  |  |  |  |  |  |  |  |
| 10 Attentional shifting | – |  |  |  |  |  |  |  |  |
| 11 Cuddliness | 0.03 | – |  |  |  |  |  |  |  |
| 12 Inhibitory control | 0.28** | 0.19** | – |  |  |  |  |  |  |
| 13 Low-intensity pleasure | 0.27** | 0.28** | 0.19** | – |  |  |  |  |  |
| 14 Activity level | −0.04 | −0.25** | −0.28** | −0.13** | – |  |  |  |  |
| 15 High-intensity pleasure | 0.04 | −0.23** | −0.14** | −0.09** | 0.37** | – |  |  |  |
| 16 Impulsivity | 0.10** | −0.24** | −0.15** | −0.10** | 0.37** | 0.25** | – |  |  |
| 17 Positive anticipation | 0.11** | 0.01 | −0.18** | 0.08 | 0.17** | 0.13** | 0.13** | – |  |
| 18 Sociability | 0.20** | −0.10** | 0.02 | 0.08* | 0.14** | 0.16** | 0.19** | 0.15** | – |

Note. *p < 0.05, **p < 0.01.

**Supplementary Table 3**  Associations of 18 subscales of the Early Child Behavior Questionnaire and expressive language scores at 40 months: regression coefficients in z-score (change in SD), 95% confidence intervals and p-values

|  | | Model 1 | | | Model 2 | | |  | Model 3 | | | | |  |  |
| --- | --- | --- | --- | --- | --- | --- | --- | --- | --- | --- | --- | --- | --- | --- | --- |
|  | | Coefficient  [95%CI] | p | | | Coefficient  [95%CI] | | p | | | Coefficient  [95%CI] | p | | |  |
| Discomfort | 0.017  [−0.078, 0.113] | | | .72 | | | −0.035  [−0.151, 0.082] | .56 | | −0.034  [−0.145, 0.077] | | | .55 | |  |
| Fear  Frustration | 0.026  [−0.006, 0.115]  −0.050  [−0.123, 0.022] | | | .57  .18 | | | 0.040  [−0.069, 0.148]  0.040  [−0.045, 0.125] | .47  .36 | | 0.022  [−0.082, 0.125]  0.035  [−0.046, 0.116] | | | .68  .40 | |  |
| Motor activation | **−0.192**  [−0.274, −0.109] | | | < .001 | | | **−0.234**  [−0.331, −0.137] | < .001 | | **−0.211**  [−0.305, −0.117] | | | < .001 | |  |
| Perceptual sensitivity | **0.144**  [0.079, 0.209] | | | < .001 | | | **0.157**  [0.079, 0.236] | < .001 | | **0.137**  [0.061, 0.213] | | | < .001 | |  |
| Sadness | −0.099  [−0.166, −0.033] | | | .003 | | | −0.072  [−0.149, 0.004] | .07 | | −0.041  [−0.115, 0.032] | | | .27 | |  |
| Shyness | 0.026  [−0.032, 0.084] | | | .38 | | | 0.026  [−0.044, 0.095] | .47 | | 0.025  [−0.042, 0.092] | | | .46 | |  |
| Soothability | **0.119**  [0.048, 0.189] | | | .001 | | | 0.028  [−0.055, 0.110] | .51 | | 0.024  [−0.054, 0.103] | | | .55 | |  |
| Attentional focusing | 0.066  [0.005, 0.127] | | | .033 | | | 0.010  [−0.052, 0.072] | .74 | | −0.006  [−0.065, 0.054] | | | .86 | |  |
| Attentional shifting | **0.232**  [0.146, 0.317] | | | < .001 | | | 0.095  [−0.005, 0.194] | .06 | | 0.087  [−0.008, 0.182] | | | .07 | |  |
| Cuddliness | 0.040  [−0.022, 0.102] | | | .20 | | | −0.014  [−0.080, 0.052] | .68 | | −0.004  [−0.066, 0.059] | | | .91 | |  |
| Inhibitory control | **0.190**  [0.115, 0.265] | | | < .001 | | | 0.110  [0.024, 0.195] | .01 | | 0.091  [0.009, 0.173] | | | .029 | |  |
| Low-intensity pleasure | **0.206**  [0.116, 0.296] | | | < .001 | | | 0.105  [0.008, 0.202] | .034 | | 0.066  [−0.026, 0.159] | | | .16 | |  |
| Activity level | −0.018  [−0.098, 0.062] | | | .66 | | | 0.076  [−0.017, 0.168] | .11 | | 0.055  [−0.034, 0.144] | | | .22 | |  |
| High-intensity pleasure | 0.021  [−0.019, 0.062] | | | .30 | | | 0.034  [−0.010, 0.077] | .13 | | 0.045  [0.003, 0.087] | | | .04 | |  |
| Impulsivity  Positive anticipation  Sociability | −0.056  [−0.108, −0.004]  0.048  [−0.028, 0.123]  0.020  [−0.015, 0.056] | | | .04  .21  .26 | | | −0.054  [−0.115, 0.006]  0.067  [−0.011, 0.144]  −0.005  [−0.041, 0.031] | .08  .09  .79 | | −0.036  [−0.094, 0.022]  0.042  [−0.032, 0.116]  0.015  [−0.021, 0.050] | | | .23  .27  .42 | |  |

Note. Model 1 = Univariate; Model 2 = Adjusted for other subscales of the Early Child Behavior Questionnaire Subscales (ECBQ); Model 3 = Model 2 with further adjustment for sex, birth weight, gestational age at birth, birth order, age of the mother, years of maternal education, annual household income, maternal history of mood/anxiety disorders. CI = Confidence intervals. Bold types represent p < .0013.

**Supplementary Table 4** Associations of 18 subscales of the Early Child Behavior Questionnaire and receptive language scores 40 months: regression coefficients in z-score (change in SD), 95% confidence intervals and p-values

|  | Model 1 | | | | | | | Model 2 | | | |  | | Model 3 | | | |  |
| --- | --- | --- | --- | --- | --- | --- | --- | --- | --- | --- | --- | --- | --- | --- | --- | --- | --- | --- |
|  | | | Coefficient  [95%CI] | | p | | Coefficient  [95%CI] | | | p | | | Coefficient  [95%CI] | | |  | p | |
| Discomfort | | −0.003  [−0.094, 0.089] | | .95 | | −0.080  [−0.194, 0.033] | | | .16 | | −0.078  [−0.187, 0.031] | | | | .16 | | | |
| Fear  Frustration | | 0.066  [−0.020, 0.153]  −0.016  [−0.087, 0.054] | | .13  .65 | | 0.064  [−0.041, 0.170]  0.044  [−0.039, 0.126] | | | .23  .30 | | 0.055  [−0.047, 0.157]  0.043  [−0.037, 0.122] | | | | .29  .29 | | | |
| Motor activation | | **−0.174**  [−0.254, −0.095] | | < .001 | | **−0.245**  [−0.339, −0.150] | | | < .001 | | **−0.225**  [−0.317, −0.133] | | | | < .001 | | | |
| Perceptual sensitivity | | **0.132**  [0.069, 0.195] | | < .001 | | **0.169**  [0.092, 0.245] | | | < .001 | | **0.150**  [0.076, 0.225] | | | | < .001 | | | |
| Sadness | | −0.039  [−0.104, 0.025] | | .23 | | −0.015  [−0.090, 0.060] | | | .69 | | 0.012  [−0.061, 0.084] | | | | .75 | | | |
| Shyness | | 0.063  [0.007, 0.119] | | .028 | | 0.057  [−0.011, 0.125] | | | .10 | | 0.054  [−0.012, 0.119] | | | | .11 | | | |
| Soothability | | 0.075  [0.007, 0.143] | | .030 | | 0.036  [−0.045, 0.116] | | | .38 | | 0.033  [−0.044, 0.110] | | | | .40 | | | |
| Attentional focusing | | 0.062  [0.003, 0.121] | | .04 | | 0.014  [−0.047, 0.074] | | | .66 | | −0.001  [−0.059, 0.058] | | | | .99 | | | |
| Attentional shifting | | **0.178**  [0.095, 0.262] | | < .001 | | 0.071  [−0.026, 0.168] | | | .15 | | 0.063  [−0.030, 0.156] | | | | .18 | | | |
| Cuddliness | | 0.028  [−0.031, 0.088] | | .35 | | −0.031  [−0.095, 0.033] | | | .34 | | −0.022  [−0.083, 0.040] | | | | .49 | | | |
| Inhibitory control | | **0.145**  [0.072, 0.218] | | < .001 | | 0.082  [−0.001, 0.165] | | | .05 | | 0.066  [−0.015, 0.146] | | | | .11 | | | |
| Low-intensity pleasure | | 0.141  [0.053, 0.228] | | .002 | | 0.041  [−0.053, 0.135] | | | .40 | | 0.008  [−0.083, 0.099] | | | | .86 | | | |
| Activity level | | −0.053  [−0.130, 0.025] | | .18 | | 0.044  [−0.047, 0.134] | | | .34 | | 0.027  [−0.060, 0.115] | | | | .54 | | | |
| High-intensity pleasure | | −0.022  [−0.061, 0.017] | | .28 | | −0.017  [−0.060, 0.026] | | | .44 | | −0.008  [−0.049, 0.033] | | | | .71 | | | |
| Impulsivity  Positive anticipation  Sociability | | −0.060  [−0.110, −0.011]  0.086  [0.014, 0.159]  0.002  [−0.032, 0.036] | | .018  .019  .91 | | −0.031  [−0.090, 0.028]  0.113  [0.038, 0.189]  −0.016  [−0.051, 0.019] | | | .30  .003  .37 | | −0.015  [−0.071, 0.042]  0.091  [0.019, 0.164]  0.003  [−0.032, 0.037] | | | | .61  .01  .88 | | | |

Note. Model 1 = Univariate; Model 2 = Adjusted for other subscales of the Early Child Behavior Questionnaire Subscales (ECBQ); Model 3 = Model 2 with further adjustment for child sex, birth weight, gestational age at birth, birth order, age of the mother, years of maternal education, annual household income, maternal history of mood/anxiety disorders. CI = Confidence intervals. Bold types represent p < .0013.
